# Steroid metabolism and hormonal dynamics in normal and malignant ovaries

**DOI:** 10.1042/EBC20240028

**Published:** 2024-12-04

**Authors:** Lucy I. Beevors, Sudha Sundar, Paul A. Foster

**Affiliations:** 1 Institute of Metabolism and Systems Research, University of Birmingham, Birmingham, U.K.; 2 Institute of Cancer and Genomic Sciences, University of Birmingham, Birmingham, U.K.; 3 Centre for Diabetes, Endocrinology, and Metabolism, Birmingham Health Partners, Birmingham, U.K.

**Keywords:** androgens, estrogens, Ovarian Cancer, Ovary, Steroid Metabolism, Steroid Sulfatase

## Abstract

The ovaries are key steroid hormone production sites in post-pubertal females. However, current research on steroidogenic enzymes, endogenous hormone concentrations and their effects on healthy ovarian function and malignant development is limited. Here, we discuss the importance of steroid enzymes in normal and malignant ovaries, alongside hormone concentrations, receptor expression and action. Key enzymes include STS, 3β-HSD2, HSD17B1, ARK1C3, and aromatase, which influence ovarian steroidal action. Both androgen and oestrogen action, via their facilitating enzyme, drives ovarian follicle activation, development and maturation in healthy ovarian tissue. In ovarian cancer, some data suggest STS and oestrogen receptor α may be linked to aggressive forms, while various oestrogen-responsive factors may be involved in ovarian cancer metastasis. In contrast, androgen receptor expression and action vary across ovarian cancer subtypes. For future studies investigating steroidogenesis and steroidal activity in ovarian cancer, it is necessary to differentiate between disease subtypes for a comprehensive understanding.

## Introduction

Steroids have a major impact on mammalian biology, influencing many processes to maintain homeostasis, regulate metabolism, and control reproduction. Normal ovarian processes, such as follicle growth, oocyte maturation, and ovulation, rely heavily on local steroid production and subsequent localized steroid-mediated signalling. Most of ovarian steroidogenesis occurs under the control of the gonadotropins FSH and LH in the granulosa and theca cells of developing and mature follicles [1]. Conversely, excessive steroidogenesis or heightened steroid signalling within the ovary may precipitate severe ovarian disorders like ovarian cancer (OC). Recently, our understanding of the intricate mechanisms governing local steroid production and its effects within the ovary have progressed significantly through various *in vitro*, *in vivo*, and clinical models. Here, we discuss these findings and examine the importance of sex steroid synthesis and action in the ovary and how this process is dysregulated in different OC types. Ultimately, understanding these processes may lead to new avenues of research and new treatments for ovarian diseases and malignancies.

## Sex steroid synthesis and metabolism in the ovary

Although it is well known that FSH regulates oestrogen synthesis via aromatase in the ovarian granulosa cell, there is limited knowledge of the expression and function of sex steroid metabolising enzymes across the ovary and their impact on ovarian function. All steroids, including the sex steroids androgens and oestrogens, are derived from cholesterol. The first step, primarily occurring in the adrenal gland, sees cholesterol converted to pregnenolone by P450scc (cholesterol side-chain cleavage enzyme) encoded by the gene CYP11A1. It has been known for decades that the ovaries are capable of *de novo* sex steroid synthesis. Indeed, more recently, CYP11A1 expression has been shown in human ovarian tissue [2]. However, in general, most active sex steroids are synthesized from circulating adrenal-derived sulfated steroid pre-cursors; sulfated steroids are water soluble and are transported freely in circulation and subsequently act as a reservoir for further downstream steroidogenesis in target cells [3]. In particular, dehydroepiandrosterone sulfate (DHEA-S), synthesized in the adrenal glands, is the main originator for active oestrogen and androgen formation.

**Figure 1:**
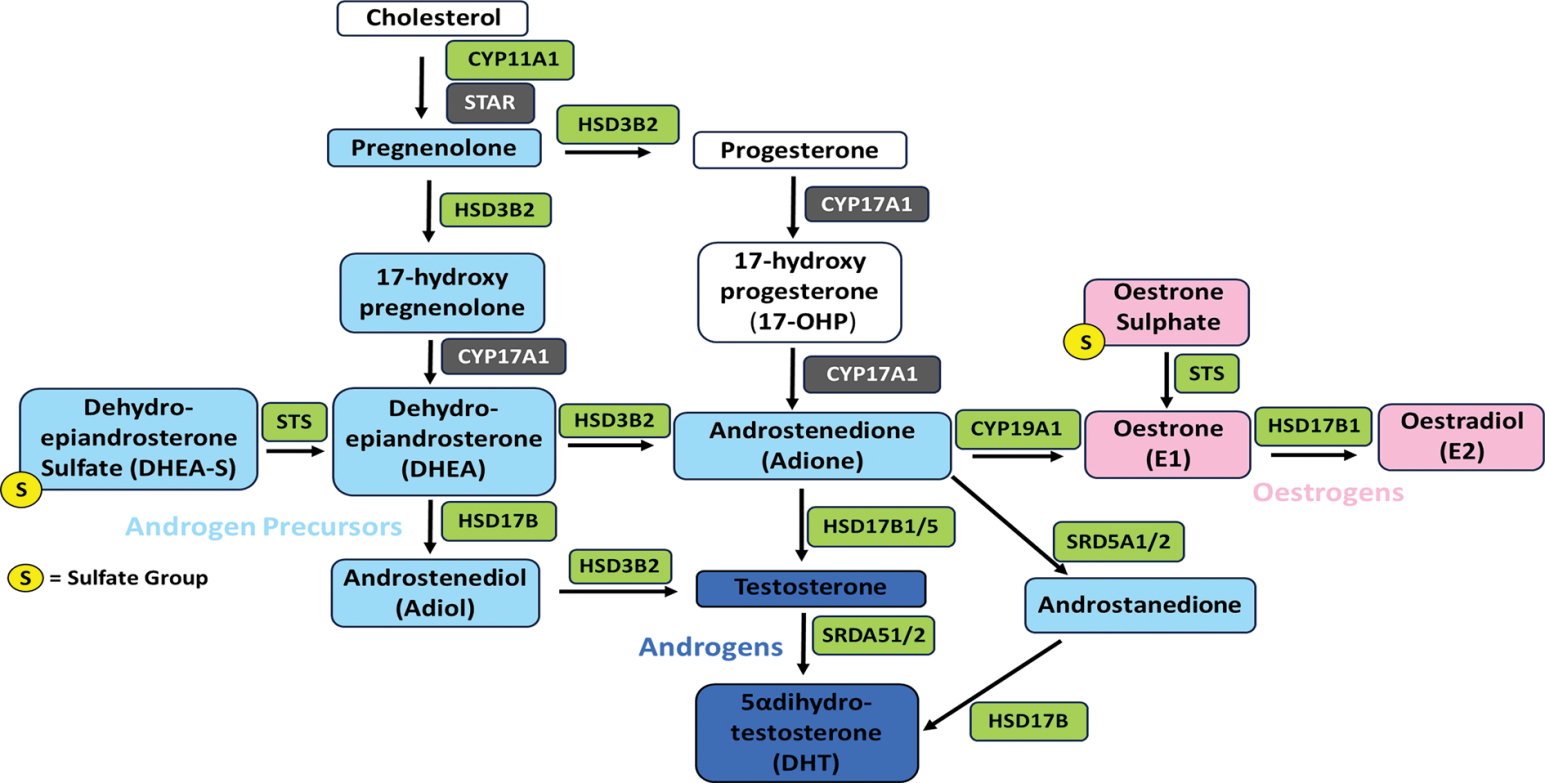
Steroid metabolism pathways and the enzymes involved (in green) involved. Active oestrogens (in pink) and androgens (in dark blue) are shown.

DHEA-S requires uptake into cells via various organic anion transporting polypeptides (OATPs) followed by initial desulfation to DHEA, from which all other steroids can be synthesised (see Figure 1) [4]. Indeed, E1-S can also be taken up into cells via OATPs and subsequently desulfated to E1, thus this pathway also acts as a source of local oestrogen synthesis (Figure 1). The key enzyme responsible for this initial desulfation step is steroid sulfatase (STS), found throughout the human body [5]. Encoded by a gene located on the distal short arm of the X chromosome in humans and other mammals [6,7], STS belongs to a family of microsomal enzymes [8] and functions to hydrolyse the sulfate-ester bonds present in sulfated steroids and sulfated steroidal precursors [7,9–11]. STS is expressed across many mammalian species and different tissues including placenta, endometrium, ovaries, prostate, testis and the adrenal gland [3,11]. Indeed, STS expression in human ovarian tissue was confirmed in early studies from the 1960s [12,13]. Human granulosa cells have abundant STS activity and may use DHEA-S as a substrate for subsequent oestradiol (E2) synthesis [14]. In human ovary tissue, STS *V*_MAX_ activity is 27.6 pmol/min/mg [15], which is comparable to the activity found in hormonally responsive human breast carcinoma (22.0 pmol/min/mg) [16]. Therefore, ovarian tissues have the potential for local steroid synthesis either *de novo* from cholesterol, via STS desulfation of DHEA-S to its deconjugated form [17] or via E1-S defulfation.

Once desulfated, DHEA can be further metabolized to androstenedione, a weak androgen, by 3β-hydroxysteroid dehydrogenase Δ^5-4^ isomerase (HSD3B). HSD3B has two isoenzymes HSD3B1 and HSD3B2, with their activity involved in the androgen, oestrogen or glucocorticoid steroidal pathways (Figure 1) [18]. The presence of HSD3B2 has been confirmed in human ovaries [19,20], where it is localized to the endoplasmic reticulum in granulosa cells, theca cells and the corpus luteum [21]. It is responsible for the oxidation and isomerisation reactions to convert the Δ5 DHEA to Δ4 androstenedione (Adione), with its activity substantially increased by FSH and decreased by GnRH [22].

**Figure 2:**
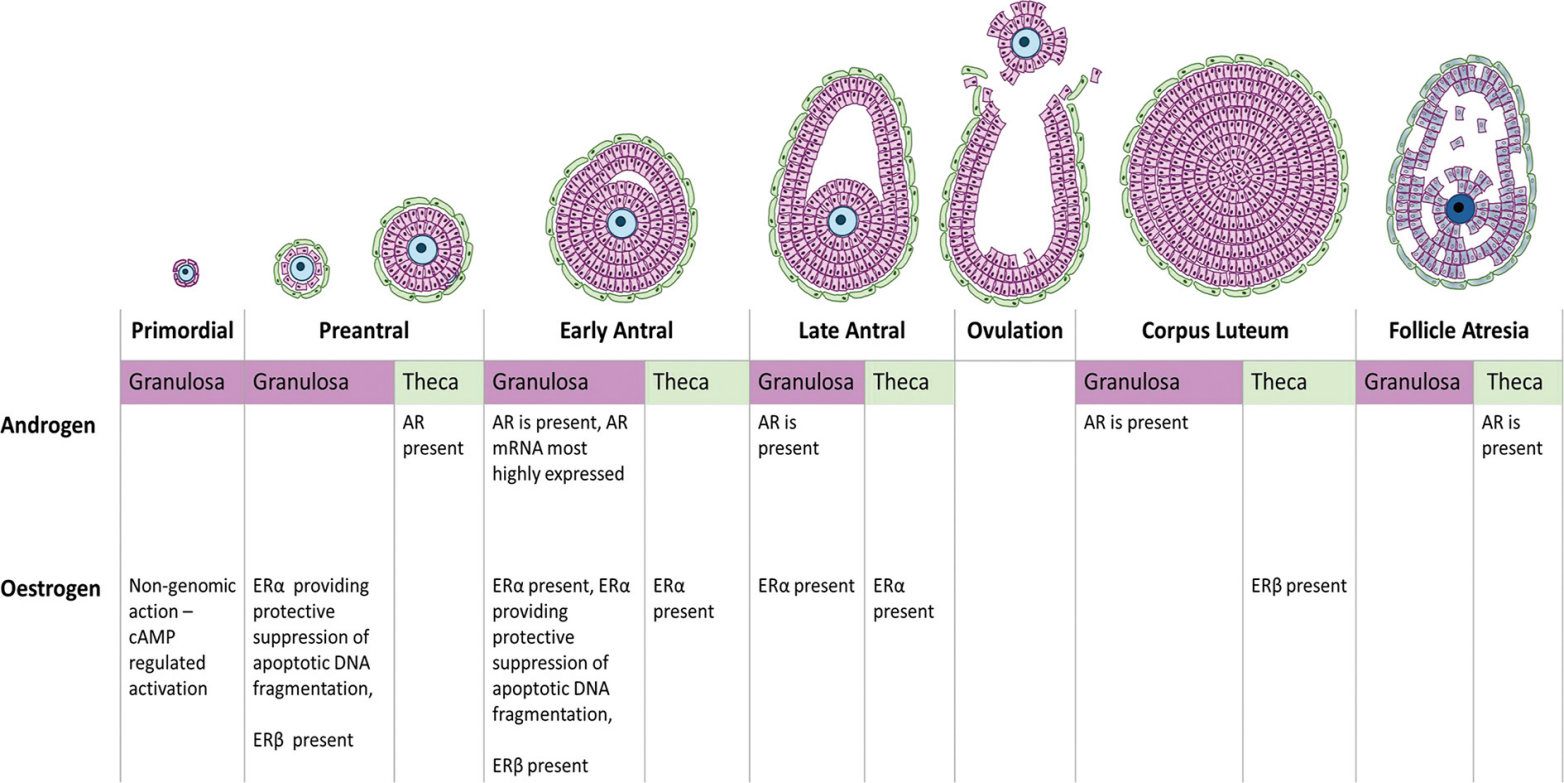
The expression of sex steroid receptors and follicle development within the ovary

Adione can be further converted to oestrone (E1) by aromatase (CYP19A1), which can also convert testosterone (T) to E2, making this enzyme essential for oestrogen biosynthesis (Figure 1). In human ovarian follicles aromatase expression levels are lowest in the granulosa cells of early follicles [23] compared with the more mature preovulatory follicles (see Figure 2). Unlike granulosa cells, thecal cells do not express aromatase and are thus unable to synthesise oestrogens, resulting in the ‘Two Cell’ model for oestrogen synthesis first suggested by Armstrong et al. in 1979 [24]. This model has become widely accepted and is well summarised in other reviews [25–28]. In postmenopausal ovaries, aromatase is predominantly expressed in epithelial rather than stromal cells [29]. With the ovarian epithelium giving rise to a large percentage of ovarian tumours, aromatase expression in these cells may provide a driver for malignancy (see section on ovarian cancer below).

17β-Hydroxysteroid dehydrogenase (HSD17B) enzymes are responsible for catalysing final steps in both the androgenic and oestrogenic steroidal pathways, including Adione to T and E1 to E2 (Figure 1). In the human ovary, oestrogenic HSD17B activity is present [30]. Indeed, HSD17B1 expression is also found outside the ovary, HSD17B1 is also present in the fallopian tube epithelium [31] and HSD17B1 mRNA is detected in the endometrium [32]. However, unlike the ‘oestrogenic’ HSD17B1 mRNA, the ‘androgenic’ isoform HSD17B3's mRNA is not found in human ovaries [30,33] despite being present in the testis [30]. Thus, it is likely that any androgenic HSD17B activity is metabolised through another HSD17B enzyme. HSD17B5 (aka. AKR1C3) is the most predominantly expressed androgenic HSD17B in human ovary [34] and has the highest catalytic efficiency for Adione reduction to T of the HSD17B enzymes [35]. However, further information on HSD17B5 expression in the ovary is limited, preventing any meaningful conclusion on its function in the tissue.

One early study concluded that T production within the human ovary occurred in healthy physiological conditions and most likely arose from the activity of HSD17B1 [36]. Since then, HSD17B1 has been shown to interact with and catalyze the conversion of androgens and oestrogens, including androstenedione and oestrogens [37,38]. HSD17B1 is localized to the cytoplasm of human ovarian granulosa cells and is also present in the corpora lutea and ovarian epithelium [36,39,40]. Levels of HSD17B1 protein and activity are significantly higher in granulosa cells incorporated in early-stage ovarian follicles [39], and are seemingly conversely correlated with aromatase expression. This pattern of enzyme expression may dictate local bioavailability of androgens and oestrogens and be integral for regulating their differing downstream effects in ovarian follicle development and maturation (see sections below on Androgen/Oestrogen Bioavailability and Action in the Ovary).

With regard androgen synthesis and subsequent action, of the classical androgen pathway, there are only two that have strong affinity for the androgen receptor (AR) and elicit downstream transcriptomic effects: T and the more potent, non-aromatisable dihydrotestosterone (DHT). Evidence suggests that DHT has twice the binding affinity for the AR compared with T [41]. T is metabolised to DHT by 5α-reductase, a steroidogenic enzyme found in two forms in humans, 5α-reductase type-1 (SRD5A1) and type-2 (SRD5A2). Currently, there is little data on expression or activity of SRD5A enzymes in the human ovary. One study suggests SRD5A2 mRNA is not present in the ovaries despite showing 5α-reductase activity was high in both human ovarian follicles and ovarian stroma at the pH preferred by SRD5A2 [42]. The lack of this SRD5A2 expression corroborates previous findings that SRDA52 was not present in foetal ovaries [43]. However, more recently evidence suggest that both SRDA51 and SRD5A2 mRNA transcripts are present in granulosa and, to a lesser degree, thecal cells, with the levels of SRDA52 higher than SRDA51 [44].

It has been known for many years that ovaries synthesis both oestrogens and androgens. More recent studies, as discussed above, highlight the more complicated enzymatic machinery that normal ovarian tissue possess to make these steroids. However, what evidence is there that ovarian tissue does this locally and how do these hormones impact normal ovarian cell function?

## Androgen bioavailability and action in the ovary

The concentrations of T and DHT in healthy ovaries are influenced by many factors, including the presence and activity of the enzymes mentioned above and the menstrual cycle. There remains a lack of studies investigating androgen bioavailability within healthy ovarian tissue; thus, it is hard to draw conclusions on their precise levels. Theca cells are a major site of LH-induced androgen production in the rat ovarian follicle [45], arguably due to these cells lacking aromatase expression. In humans, theca cells within the ovarian stroma secrete higher levels of androgens than oestrogens; secreting 50 µg/day of T, accounting for approximately 25% of circulating T levels in serum [46,47]. Other sources suggest androgen production exceeds oestrogen synthesis, with the ovaries together producing 0.8−2.8 mg/day of Adione, 0.06−0.10 mg/day T and 0.3−3.0 mg/day of DHT [48–50]. Furthermore, higher androgen concentrations are most prominent in cells involved in early follicles [51] and as such there is not only inter-cell type variability of androgens but also intra-cell type differences between follicles at various developmental stages.

However, these androgens will require AR expression to have any local impact. AR mRNA and protein are both present within granulosa cells of murine ovarian follicles [52,53]. AR is expressed as early as Day 17 postpartum in prepubertal mice [53] and has also been identified in the granulosa cell nuclei of early antral primate follicles [54]. In human ovaries, AR mRNA is expressed during follicular development [23,55] and is most highly expressed in the granulosa cells of small antral follicles compared with those more developed [23] (Figure 2). AR protein is present in ovarian stromal cells at all distances from follicles [56,57], in the thecal cells of pre-antral and atretic follicles [57], in the granulosa cells of antral follicles, and the corpus luteum of normal ovaries [56]. AR protein was consistently present in ovarian cells but varied in intensity across the menstrual cycle [58]. Furthermore, AR is also present in the ovarian surface epithelium (OSE) [59], which indicates a potential driving pathway for proliferation in OSE and other cell types.

However, with normal ovaries having the potential for local androgenesis and AR expression, how do androgens affect their function in healthy ovaries? Upon treatment with DHT, translocation of the AR to the nucleus of murine follicular cells is significantly increased compared to controls [53], allowing for potential AR-mediated transcriptional changes in these cells. Moreover, T induces the maturation of meiosis-arrested murine oocytes [60] and active androgens increase their growth and diameter without any E2 production [53,61]. One study demonstrated T-mediated MAPK signalling and subsequent phosphorylation of p42/p44 was reduced by an AR antagonist yet were largely unaffected by an aromatase inhibitor [60]. Therefore, the T-induced growth and maturation of murine oocytes may be regulated through MAPK signalling pathways. In primary OSE cultures, treatment with a synthetic androgen (Mibolerone at 1 nM) caused significant increase in DNA synthesis in 55% of cultures and a decrease in cell death in 40% of cultures [62]. Furthermore, in granulosa cells of primate ovaries, expression of AR mRNA was positively associated with expression of Ki-76, a proliferation marker, and inversely correlated to apoptosis [63]. Additionally, a knockdown of AR in murine models showed reduced fertility [64].

**Figure 3:**
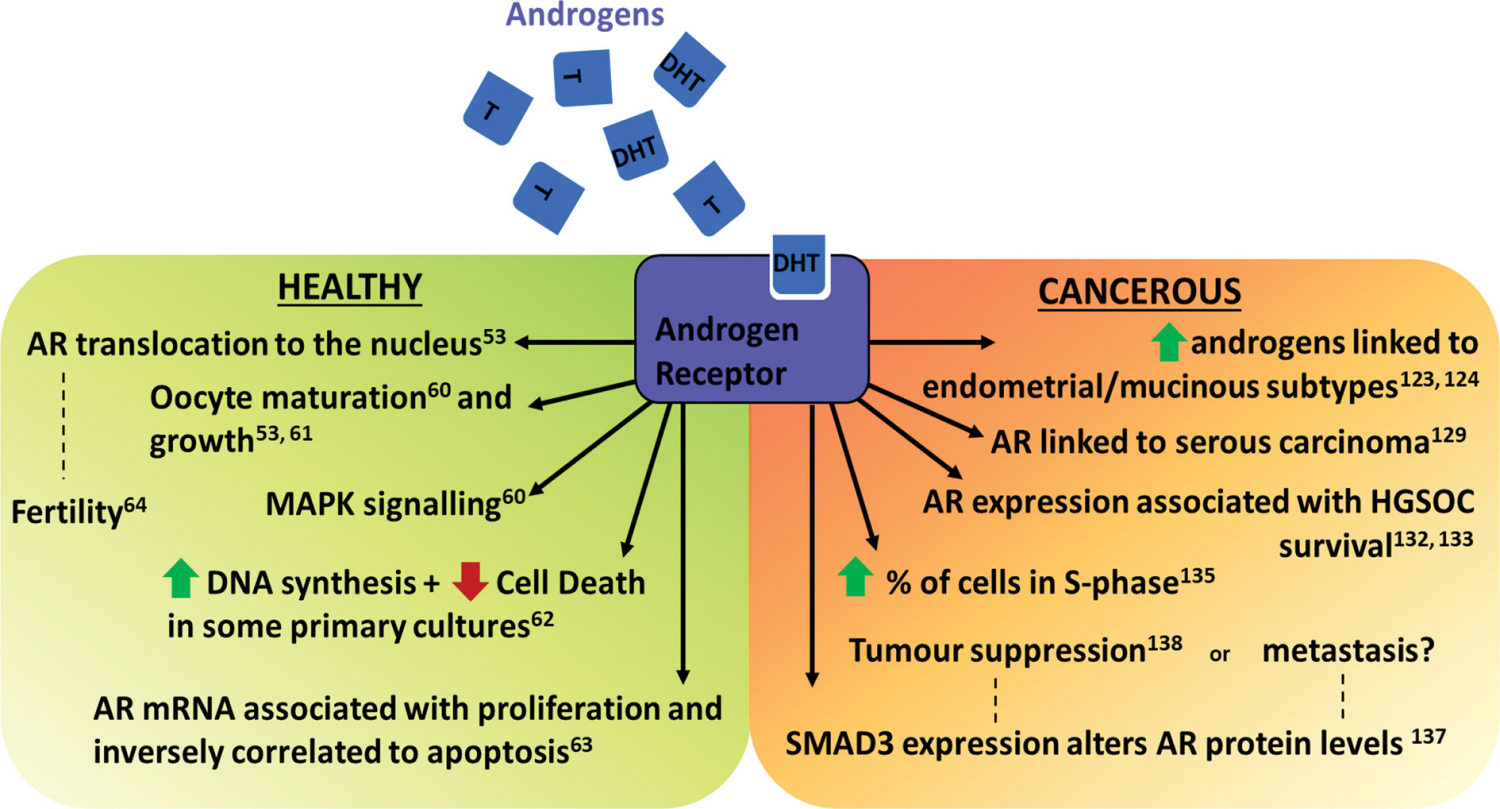
Overview of the actions of androgens T and DHT on healthy and cancerous ovarian tissue

These findings (summarised in Figure 3) imply that androgens are involved in driving proliferation and hindering apoptosis in multiple parts of the ovary in animal models and may play a role in their ultimate fertility. Furthermore, their mitogenic role in normal ovarian cells makes them potentially suspicious factors in ovarian malignancy development (see section below on Ovarian Cancer)

## Oestrogen bioavailability and action in the ovary

It is widely acknowledged that in premenopausal females the ovaries are the main site of oestrogen synthesis and subsequently the largest contributor to circulating oestrogens. Thus, some have suggested the ovaries are continuously saturated with oestrogens [65]. However, oestrogen concentrations in normal ovaries are not fully elucidated. Studies often focus on circulating oestrogen concentrations and oestrogen secretion rates from the ovary. Reference values are established for normal cycling women’s serum levels of E2 at each stage of the menstrual cycle, having the highest median value of 671.06 pmol/L (a 5−95% range of 482.0−1517.7 pmol/L) during the LH peak (Day 0), and the lowest reference median of 149.74 pmol/L (5−95% range of 57.5−194.4 pmol/L) in the early follicular phase (Days -15 to -6) [66]. Unsurprisingly, ovarian oestrogen secretion also fluctuates; during the late follicular stages (the peak for oestrogen secretion) the ovaries together secrete 0.25−0.50 mg/day of E1 and 0.4−0.8 mg/day of E2 [48–50], although a recent review suggests much lower values between 70 and 500 μg E2 secreted daily [65].

The effects of oestrogens are regulated by the oestrogen receptors (ERs), ERα and ERβ. In rat ovaries transcripts of both types are expressed; ERα mRNA is present in all cell types whereas ERβ mRNA is typically localised to the granulosa cells of maturing follicles and at low levels in the corpus luteum [67]. Furthermore, ERα protein expression is more common in theca cells whilst ERβ protein is consistent with its mRNA expression and is mainly localised to the granulosa cells of follicles [68,69]. In primates, both ERα and ERβ mRNA is present in granulosa cells with ERα mRNA levels altered by progesterone [70], demonstrating the reciprocal influence between steroid levels and receptor levels.

In primary samples of normal human ovaries, ERα and ERβ mRNA transcripts appear present at a consistent level irrespective of menopausal status; however, only 10 samples were obtained for the present study suggesting further evidence is required to confirm their presence [71]. Furthermore, primary cultures of normal human ovarian surface epithelium (HOSE), demonstrated co-expression of ERα and ERβ mRNA [72,73], with other variants containing exon-deletions also commonly present [73]. Though ERβ mRNA expression in human corpus luteum is generally conserved, there are contradictory findings on the expression of ERα mRNA [74,75]. In terms of protein expression, human foetal ovaries are documented to consistently express ERα and ERβ in their granulosa cells and oocytes from 26th to 20th week of development onward, respectively [76]. In adult ovaries, during follicle development, granulosa cell, thecal cells, surface epithelial and stroma all express ERβ [77,78]. However, ERα is detected in germinal epithelial cells, antral follicles and the interstitial gland [77,78]. ERβ is also localised to the steroidogenic cells in the corpus luteum [79].

**Figure 4:**
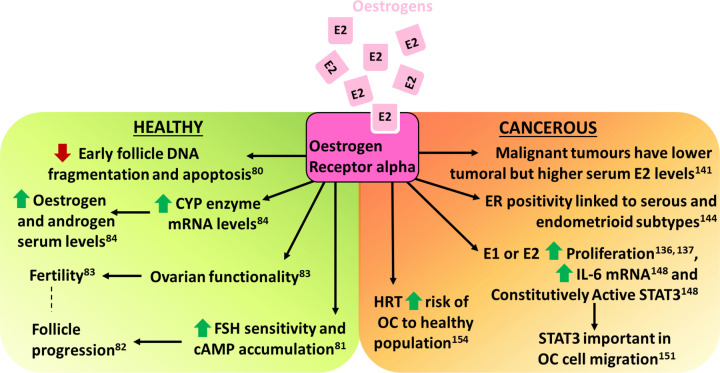
Overview of the actions of oestrogen E2 on healthy and cancerous ovarian tissue

Oestrogens are implicated in follicle atresia demonstrated by the influence of a synthetic oestrogen on DNA fragmentation as a conduit for apoptosis in rat ovaries [80]. Granulosa cells in preantral and early antral follicles had increased apoptotic DNA fragmentation following removal of synthetic oestrogens, which was ablated after reintroduction of the synthetic oestrogen or replacement with E2 [80]. This suggests oestrogens have a preservative effect in early follicles. Thus, complementing the hypothesis that oestrogens stimulate proliferation in granulosa cells of small follicles [65] and enhances sensitivity of these cells to FSH thus increasing cAMP accumulation [81]. In murine ovaries, increased cAMP levels resulted in elevated primordial follicle activation while reduced cAMP levels inhibited follicular activation [82]. This implicates oestrogens in the progression of ovarian follicles and fertility. In fact, female mice lacking ERα demonstrate impaired ovarian function which contributes to their infertility and ERβ lacking mice have ovarian defects causing subfertility [83]. Furthermore, ERα knockout mice expressed elevated levels of CYP17 and CYP19 mRNA in the ovary and the same mice showed elevated levels of Adione and E2 in serum [84] demonstrating the relationship between ER expression, steroidogenesis enzymes, steroid levels and ovarian function. Oestrogen action in early ovarian follicular maintenance and follicular activation in the normal ovary is summarised in Figure 4.

## Ovarian cancer

Ovarian cancer (OC) is the sixth most common cancer among UK females with approximately 7,500 newly diagnosed cases per year [85]. OC is the most lethal gynaecological cancer, killing more women than womb, cervical, vaginal and vulval cancers combined [86]. Unfortunately, OC is often diagnosed at advanced stages and presents a clinical challenge due to the heterogeneity of the disease, with findings from a discussion between an international panel of researchers highlighted the necessity for discrimination between different kinds of OC [87]. OCs are categorised into epithelial ovarian cancers (EOC) and non-epithelial ovarian cancers (NEOC). NEOCs account for 10% of OC cases comprising heterogeneous subgroups largely consisting of germ cell tumours (GCT, arising from primodrial germ cells) [88], and sex-cord stromal tumours (SCST, originating from the ovarian stroma and sex cord) of which granulosa cell tumours (GrCT) are most frequent. NEOCs comprise some extremely rare tumour types including small cell sarcomas (0.01%) [89]. SCSTs are usually sex hormone producing tumours, often producing both oestrogens and androgens and are usually granulosa cell tumours [90].

Since the turn of the century, EOCs have categorised as type-1 tumours (clear cell, endometroid, mucinous, seromucinous, Brenner and low-grade serous tumours) and type-2 tumours (high grade serous, undifferentiated carcinoma, carcinosarcoma) (see Table 1) [91]. In general, the type-1 tumours were described as indolent and high-grade serous ovarian cancers (HGSOC) as more aggressive type. Recently, the type 1/type 2 classification has been criticised for its generalisation and lack of accuracy and specificity, particularly for grouping type-1 tumours together despite distinct molecular and etiological differences [87,92]. This dualistic system is still in use. However, there is a push in the field to better characterise each subtype into further subgroups based on their clinical characteristics, histology, mutation profile and gene expression, with the TCGA identifying four clusters in HGSOC gene expression analysis [93]. This drive to more accurate classification beyond type-1 and type-2 is necessary for advancements to be made in patient diagnosis and treatment. Indeed, despite being labelled as indolent, type-1 tumours, when diagnosed at advanced stages are often resistant to platinum-based chemotherapy resulting in a poorer 5 year prognosis than the ‘aggressive’ HGSOC tumours [94].

**Table 1:** Histological subtypes of epithelial ovarian cancer with associated information

Epithelial ovarian cancer subtype	∼% of total EOC diagnosis	Origin	Associations	Most common genetic mutations
**Endometrioid**	8%	Endometriosis, Ciliated cells	Endometriosis	CTNNB1, PTEN, PIK3CA, ARID1A
**Clear cell carcinoma**	10%	Endometriosis, Secretory cells	Endometriosis	PIK3CA, ARID1A
**Mucinous**	3%		Often metastases from other tissues	KRAS, BRAF, ERBB2, TP53
**Low grade serous** (Type 1 - more genetically stable, more commonly diagnosed at early stages)	5%	Ovarian surface epithelium, Fallopian tube	Endometriosis	KRAS, BRAF, ERBB2
**High grade serous carcinoma** (Type 2 - more genetically unstable, commonly diagnosed at later stages)	70%	Distal fallopian tube, Ovarian epithelium	Basal-like breast carcinoma	TP53, CCNE1

## Sex steroid metabolism in ovarian cancer

As previously discussed in this review, sulfated sex steroid hydrolysis by STS is the starting point of both androgen and oestrogen synthesis. One early study investigating DHEA-S to DHEA and E1-S to E1 conversion in lower reproductive tract carcinomas (including squamous cell vaginal, ovarian carcinomas and endometrial adenocarcinomas) suggested that STS driven steroid synthesis may be responsible for steroid synthesis in gynaecological carcinomas [95]. Indeed, in HGSOC cell lines, STS expression is high and E1-S can be metabolised to E1 and E2, although the concentration and velocity of oestrogen production from E1-S appeared inversely proportional to chemoresistance [96]. Another study showed a significant difference in oestrogen metabolism between platinum-sensitive and -resistant HGSOC cell lines, with sensitive cells exhibiting up to a 60-fold increase in steroid hormone formation, which could be reversed with interleukin-6 (IL-6) treatment [97]. Indeed, IL-6 treatment lowered E1 (and DHEA) metabolism to other metabolites, such as E1-S or E2, suggesting inflammation as a regulator in oestrogen metabolism in ovarian cancer. Thus, taken together, the role of STS in the maintenance and proliferation of ovarian tumours is unclear and needs further investigations.

STS presence in OC differs based on subtype; it is present in approximately 70% of ovarian clear cell carcinomas, 33.3% of serous and 50% of mucinous OC [98]. Indeed, patients with papillary tumours had both an increased staining of STS and a significantly lower survival rate compared to those diagnosed with solid tumours [98]. This implies the tumourigenic potential of STS in ovarian tissue, most likely linked to local steroid synthesis. Interestingly, STS activity is six times greater in the OC cell line OVCAR-3 compared with the hormone-responsive breast cancer cell line MCF7 [99]. However, this increase is not directly associated with elevated STS mRNA expression. In EOC patients, STS protein expression was not linked to tumour histology or FIGO stage but was present in 65% of patients studied (including serous borderline, low grade serous, HGSOC, mucinous and endometrioid) [100]. Moreover, in these patients, STS expression was significantly associated with reduced survival [100]. AR expression alone did not show the same effect on patient survival, however, when AR+ tumours also express STS, this significantly reduces patient overall survival and yields an ‘independent predictor of poor prognosis’ [100]. STS has also been associated with oestrogens in OC. Indeed, high levels of STS activity were found in benign and cancerous ovarian tumours alongside a significant positive correlation between serum E2 levels and the conversion of E1-S to E2 [101]. However, this study had a very small sample size and results will need to be corroborated by a larger study to discriminate between benign/malignant and different subtypes and classifications of tumours. In HGSOC samples, SULT1E1 (an enzyme that metabolises the inverse oestrogenic reaction of STS) expression was linked to increased survival [102]. Thus, desulfation in EOC potentially correlates to active androgen and oestrogen synthesis, thus impacting the progression of this disease.

Despite >81% of primary tumours expressing aromatase, malignant and metastatic ovarian tumours have lower aromatase expression and activity levels compared with healthy ovarian tissue and ovarian cysts [103,104]. There is no significant correlation between aromatase activity and cytosol oestrogen content or ER [103,104]. Indeed, there is no significant differences in aromatase expression in different tumour grades, stage or survival and survival is not linked to aromatase activity [29,105]. Therefore, it is unlikely that aromatase is involved in OC development and progression. This suggests that it is either local oestrogen synthesis via STS or uptake of circulating androgens or both that impact OC development, survival, and proliferation.

As previously discussed, OSE cells express HSD17B1 [40]. In HGSOC, the expression of HSD17B1 protein is associated with an increase in patients’ overall survival [106] although across OC HSD17B1 expression is far less common (10%) than ‘oestrogen-inactivating’ HSD17B's (86%) [107]. Indeed, HSD17B1 expression is higher in type-1 tumours than type-2 [108], and is associated with a less aggressive phenotype. This may imply that HSD17B1 activity in oestrogen synthesis is not driving the majority of OC malignancy.

HSD17B3 is not expressed in the human ovary and thus the conversion of Adione to T might be catalysed by HSD17B1 or HSD17B5. However, HSD17B5 expression was significantly lower in EOC than OSE [34] and as previously discussed HSD17B1 is unlikely to be driving aggressive disease. Furthermore, potent inhibitors of HSD17B5 exhibited superior anti-proliferative and anti-migration effects on a chemoresistant ovarian cancer cell line compared with carboplatin, with these effects similar to cisplatin [109]. This suggests that changes in androgen synthesis in OCs may utilise this pathway for growth. However, other HSD17Bs may also be involved. For example, HSD17B12 functions as a reductase similar to HSD17B1 [38]. It has high sequence similarity with HSD17B3 [110] and has a 5% metabolism rate for Adione [110]. Interestingly, the murine equivalent to HSD17B12 catalyses Adione to T [111]. Indeed, ovarian cystadenocarcinomas, adenomas, and carcinomas of mixed histological types showed a general trend, with a significant increase in HSD17B12 observed in more aggressive forms, particularly between low-grade and high-grade endometrioid adenocarcinomas [112]. This corroborates an earlier finding that in patients with mixed EOC tumour subtypes and FIGO stages, a heterogenous or weak/moderate intensity of HSD17B12 correlates with longer overall survival and time to first recurrence [113]. However, further research into the presence and function of HSD17B's within OC cell lines and primary tumours is required to further elucidate the importance of these pathways.

To synthesis the most active androgen, DHT, SRD5A enzymes are required. Case control studies in stage IV epithelial OC patients show no significant correlation between SRD5A2 immunoreactivity and AR labelling index [114] and that a previously implicated allele is not associated with alterations in OC risk [115]. Similarly, in white non-Hispanic patient populations there is no association between SRD5A1 and OC risk [116]. In contrast, other studies suggest SRD5A2, specifically the SRD5A2 V89L SNP, is associated with an increased risk of epithelial OC in Caucasian populations [117]. With OC being so heterogenous, further understanding of steroidogenesis enzymes and AR signalling on the development and progression of different subtypes EOC is required [118].

## Androgen bioavailability and action in ovarian cancer

Decades ago, Risch put forward the idea that androgens may be involved in the development of EOC via stimulating epithelial cell proliferation [48]. Early clinical studies showed mixed results on serum androgen concentrations and associated OC risk. For example, androgen levels, especially in the case of DHEA, in women who developed OC were significantly lower than matched controls [119]. In contrast, others show that compared with controls, OC patients have significantly higher Adione and DHEA concentrations [120]. Indeed, sulfated precursors may also be associated with OC risk as another cross-sectional study demonstrated a borderline significant association between DHEA-S levels in postmenopausal women and a familial history of OC (*P*=0.05) [121]. Despite stating their data does ‘not support the hypothesis that androgen-related disorders increase the risk of OC’, one study showed women who had ever taken a T supplement were at a significantly increased risk of developing EOC [122]. Thus, the risk of OC may increase with higher circulating androgen concentrations, complementing Risch’s initial hypothesis. However, further studies in the field must differentiate between subtypes and histological groups of EOC to allow for more accurate and clinically relevant conclusions.

Unfortunately, these early investigations treated OC as a uniform disease which may impact interpretation as the role of androgens in OC development is potentially subtype specific. One recent study comparing 565 OC cases with 1,097 matched controls showed Adione concentrations were significantly higher in patients with mucinous type tumours and significantly lower in patients with serous tumours, compared with matched controls [123]. Indeed, a doubling of Adione in OC patients carried a 99% increase in grouped low grade, endometrial and mucinous tumours [123]. Furthermore, T positivity is associated with higher overall risk of EOC development and, similarly to Adione, higher T is associated with higher risk of endometroid and mucinous tumours [124]. This implies different OC subtypes have different androgen synthesis capabilities and differing responses to androgen signalling. Taken together the above studies tentatively show some association between androgen and androgen precursors levels and the subtype specific OC risk. Further epidemiological, clinical and *in vitro* studies are required to fully elucidate the potential role of androgens in OC carcinogenesis.

Multiple early patient studies demonstrated widespread nuclear AR expression in primary OCs (from 43.7% to 90% of tested tumours) [125–130]. Though, some borderline epithelial tumours were also found to be AR positive [125,126] as were a high proportion of benign OCs [131]. More recently, AR was most highly expressed in the serous carcinoma subtype [129]. Moreover, despite AR not being a prognostic factor in all OC subtypes, for HGSOC, AR expression is associated with prolonged disease specific survival [132]. AR expression is also significantly associated with increased progression free survival [133]. Similarly to androgen levels, the presence and activity of AR in OC appears to besubtype dependent.

Androgens are also potentially involved in the proliferation and progression of OC. Animal models have shown that T treatment increases cystadenomas and papillomas and enriches epithelial cysts [134]. In human HGSOC primary OC cultures, DHT increases cellular division by increasing the proportion of cells in S-phase compared with controls [135]. There is a strong correlation between S-phase fraction changes and nuclear AR expression. Indeed, responsive cells treated with anti-androgens decrease S-phase cycle [135]. In OC cell lines (OVCA 420, OVCA 429, OVCA 432, and OVCA 433, all late-stage serous ovarian adenocarcinomas), DHT and T increase proliferation (2- and 4-fold enhancement) compared with controls, though this was a reduced response compared with normal surface epithelium and immortalized normal surface epithelium [136]. The processes through which androgen stimulation and AR transcriptional activity alters OC are still being investigated. One recent study using serous OCVAR-3 and non-serous SKOV3 and ES2 cell lines, investigated DHT treatment and SMAD3 overexpression or silencing on AR protein levels [137]. Overexpression and silencing of SMAD3 decreased AR levels suggesting a dose dependent interaction between these proteins [137]. SMAD3 is well known to play a role in TBF-β mediated immune suppression and promoting transcriptional changes linked to metastasis. However, SMAD3 may function as a tumour suppressor by inducing apoptosis and inhibiting cellular proliferation [138].

High androgen levels appear to increase patient risk of developing OC, particularly the endometrial and mucinous subtypes. However, the molecular mechanisms behind this are poorly understood and the precise role of androgens in OC development requires further elucidation. These conclusions are summarised in Figure 3. In the future, it will be essential to investigate androgen levels and action within individual subtypes of EOC to effectively understand this heterogenous disease.

## Oestrogen bioavailability and action in ovarian cancer

As with androgens, the relationship between oestrogens and OC risk among patient populations has yielded conflicting results. UK Biobank data revealed a notable association between elevated blood concentrations of E2 and an increased risk of OC (OR = 3.18, [95% CI: 1.47−6.87], *P*=0.003) [139]. However, this analysis was confined to categorizing participants as either ‘high oestradiol’ with ≥175 pmol/L or ‘low oestradiol’ <175 pmol/L. Conversely, a larger-scale US study found no association between E2 levels and OC risk [140]. However, a positive association between E1 levels and OC risk (OR = 1.54, p-trend = 0.05) has been identified, though no statistical significance was observed between extreme quintiles (Q5 vs Q1: 1.54 (0.82−2.90) [140].

When investigating OC, it is crucial to consider oestrogen levels within both serum and tumour tissues as postmenopausal women’s normal or neoplastic ovarian tissues exhibit median concentrations ∼100 times higher (ranging from 9.25 to 16.44 pmol/g) than serum levels (ranging from 0.07 to 0.10 pmol/g), and while tumoral E2 concentrations were lower in borderline and cancerous tumours than normal or benign, the opposite was true for serum concentration levels [141]. Surgical removal of tumours saw a marked decrease in serum E2 concentrations, leading the authors to propose that the increased E2 was synthesized *de novo* in the ovarian tumour. Notably, cancerous tumours exhibited significantly lower levels of E2 compared with benign tumours [141]. Variations in circulating E2 levels were observed between type-1 and type-2 tumours and among different FIGO stages [141]. A case study illustrated E2 levels outside reference ranges in serum from a postmenopausal OC patient [142]. However, there is often variability in reference ranges used. Collectively, these data suggest postmenopausal ovarian tumours synthesis high levels of oestrogens and augment circulating oestrogen levels.

Oestrogen receptor expression has been shown in healthy ovaries, but what is the ER status of ovarian cancers? Studies on primary cultures (OVCA420, OVCA429, OVCA432, and OVCA433), and established OC cell lines (DOV13, SKOV3, and CAOV3) show consistent ERα mRNA expression, with levels akin to human ovarian surface epithelial (OSE) cells [73]. However, within distinct classes of OCs, the expression of these receptors may diverge. Malignant tumours typically exhibit higher levels of ERα mRNA compared with benign tumours, while the opposite trend holds true for ERβ [65], suggesting potential differential involvement of these receptors in OC progression. Indeed, within advanced HGSOC, high activity of the ER was associated with favourable disease free and overall survival of postmenopausal patients [143].

The percentage of ER-positive immune-stained cells appears to correlate with tumour subtypes, with >85% expression observed in serous and endometrioid compared with <19% in mucinous and clear cell [144]. Interestingly, in matched relapsed high-grade samples, there was no significant difference in ER expression levels [145]. Given the heterogeneity of OC, this relationship is likely subtype specific. Indeed, another study noted a significant increase in the percentage of cells expressing ERα and ERβ in recurrent granulosa cell tumours (from 24% to 38%) [146].

Oestrogens have also been implicated in the aetiology of OC, with some human OC cell lines (including PE01, PE04, PE06, OVCA 420, OVCA 429, OVC A432, and OVCA 433) showing increased proliferation when treated with E1 or E2 [136,147]. Similarly, treating both human ovarian surface epithelial (HOSE 301, HOSE 306, HOSE 642, and HOSE 12-12) and OC cell lines (OVCA 420, OVCA 429, OVCA 432, and OVCA 433) with E2 or E1 leads to approximately 1.5- and 3-fold increases, respectively, in IL-6 mRNA levels compared with controls [148]. Moreover, IL-6 induces the phosphorylation of STAT3, while in these OC lines, STAT3 phosphorylation is constitutive and not dependent on IL-6 [148].

STAT3 is a transcription factor that remains constitutively active in many cancers and regulates the expression of genes involved in proliferation, metastasis, metabolic reprogramming, and chemoresistance [149,150]. Indeed, studies utilizing siRNA to knockdown STAT3 expression significantly reduced OC cell migration capabilities compared with controls [151]. In a study on breast cancer, IL-6/STAT3 could utilize oestrogen response elements (ERE) and act independently of ER/FOXA1 to drive distinct oncogenic transcriptional actions [152]. This suggests that STAT3 action may involve oestrogen crosstalk or act independently to drive oncogenicity.

The differential roles of ERα and ERβ in OC progression, as suggested by their expression levels, extend to their downstream effects. Expression of ERβ1 in SKOV3 cells led to reduced cell proliferation, enhanced apoptosis, and decreased motility. This was accompanied by an up-regulation of cyclin-dependent kinase inhibitor p21 and a down-regulation of cyclin A2 mRNA [153]. In primary granulosa cell tumours, a 24-h treatment with E2 resulted in a slight yet significant decrease in caspase-3/7 activity. After 72 h, there was a notable increase in the size of the cell population [146].

The utilization of hormone therapy appears to have a discernible impact on the incidence of OC in both patients and the broader female population. A meta-analysis revealed that current users of oestrogenic hormone therapy, regardless of duration, faced an elevated relative risk of developing OC [154]. Even in prospective studies involving postmenopausal women who had used hormone therapy for less than 5 years, the relative risk of OC was significantly increased (RR: 1.43, 95% CI: 1.31–1.56; *P*<0.0001) [154] Likewise, the current or recent use of oestrogen or oestrogen-progesterone medications was associated with increased relative risks for serous (RR: 1.53, 95% CI: 1.40–1.66; *P*<0.0001) and endometrioid (RR: 1.42, 95% CI: 1.20–1.67; *P*<0.0001) tumour types [154].

However, a small Phase III randomised controlled trial investigating adjuvant hormone therapy (AHT) in OC patients found AHT may have a beneficial effect and promote patient survival. Patients receiving AHT demonstrated improved overall survival compared to controls (hazard ratio: 0.63; 95% CI: 0.44–0.90; *P*=0.011), as well as enhanced relapse-free survival (hazard ratio: 0.67; 95% CI: 0.47–0.97;* P*=0.032) [155]. However, this trial was closed prematurely due to challenges with recruitment, limiting the conclusions that can be drawn.

Evidently, there exists data indicating a correlation between oestrogen action in cellular models of OC and the risk and incidence observed in patients (summarised in Figure 4). Nevertheless, further research is warranted to comprehensively elucidate the intracellular mechanisms involved. In particular, understanding the differences in AHT action in a healthy population compared to OC patient groups.

## Targeting sex steroid metabolism in ovarian cancer

Chemotherapy is recommended for stage 1C epithelial ovarian cancer, including serous cancer, following surgery. For latter stages of disease, cytoreductive surgery should be followed by platinum-based chemotherapy. However, low-grade serous ovarian cancers have low response rates to this form of chemotherapy [156]. One study of 36 women with low-grade serous ovarian cancer who received neoadjuvant chemotherapy and were matched to patients with high-grade disease, only 4 (11%) had a partial response compared with 27 (75%) women with high-grade [156]. Thus, endocrine therapy is not generally used to treat high-grade serous ovarian cancer. However, it is widely used for managing low grade serous ovarian cancers, showing effectiveness in various clinical settings, such as primary adjuvant, maintenance, and salvage therapies [157,158]. Approximately 70% of LGSC cases exhibit high ER expression, and 30% show high PR expression, making them potential targets for treatment [159]. However, no direct link between receptor expression and therapy response has been established [160]. A retrospective analysis reported a 9% objective response rate and 61% disease stabilization [161]. In the PARAGON phase II study of anastrozole, around 60% of women showed clinical benefit at 6 months, with 14% showing partial responses [158]. Fader et al. studied hormonal monotherapy (letrozole, anastrozole, or tamoxifen) after cytoreductive surgery in 27 women with stage II-IV low grade serous ovarian cancer. Preliminary results showed similar survival outcomes compared with those treated with surgery and chemotherapy, suggesting chemotherapy might be unnecessary for advanced-stage patients receiving adjuvant hormonal therapy [157].

Using hormonal therapy in the treatment of ovarian cancer may be associated with several potential complications largely due to the systemic effects of altering hormone levels. A principal concern revolves around the skeletal system; adjuvant hormonal treatments can disrupt the oestrogen-skeleton axis, leading to decreased bone mineral density, heightened risk for osteoporosis, and increased susceptibility to skeletal fractures [162]. Such treatments may induce primary ovarian failure in premenopausal women, causing lower levels of circulating oestrogen and consequently, osteopenia. Furthermore, adherence to hormonal therapy regimens is significantly challenged by adverse side effects. Studies on breast cancer patients undergoing hormonal therapy demonstrate that treatment-related side effects, such as musculoskeletal/joint pain, can profoundly impact adherence [163].

## Conclusions

Ovarian cancer is a heterogeneous, lethal disease that appears heavily influenced by androgen and oestrogen action. As such, the enzymes involved in the steroidogenesis pathways, such as STS, are crucial factors to investigate so that we may elucidate their individual roles in the ovary and in ovarian carcinogenesis. In healthy ovaries, both androgens and oestrogens drive ovarian follicle activation, development and maturation. However, in ovarian cancers, STS may be associated with more aggressive forms of OC while androgens and the AR may have distinct action in different subtypes of OC. Thus, future studies investigating the role of androgens within specific subtypes of OC are required to further elucidate their differing concentrations and downstream action. Oestrogens are involved in OC risk and proliferation, potentially through IL-6 and STAT3 pathways with ERα particularly associated with malignancy and HGSOC. Due to the differing associations seen between type 1 and type 2 OCs and hormonal steroids, it is crucial for future studies in the field to distinguish between subtypes of OC to faithfully address the disease, identify potential therapeutic targets and allow for subtype specific treatments for patients.

## Summary

- Healthy ovaries have enzymatic machinery for both *de novo* and sulfated precursor derived steroidogenesis, including STS.
- Across species, in the ovary, androgens appear to mediate DNA replication and cell death, AR nuclear translocation, oocyte maturation and growth potentially via MAPK signalling which may impact fertility.
- High androgen levels seem associated with an increased risk of endometrioid and mucinous tumour subtypes yet AR expression is most common in HGSOC and may be protective.
- Oestrogens are involved in supressing follicular cell apoptosis, promoting ovarian function and response to and production of steroid hormones.
- In ovarian cancers, oestrogens elicit increased proliferation and oestrogenic HRT appears to increase OC risk in the population.

## Abbreviations

AHT: adjuvant hormone therapy
AR: androgen receptor
EOC: epithelial ovarian cancers
ERE: oestrogen response elements
GCT: germ cell tumours
HGSOC: high-grade serous ovarian cancers
HSD17B: 17β-Hydroxysteroid dehydrogenase
IL-6: interleukin-6
OATP: organic anion transporting polypeptide
OSE: ovarian surface epithelium
STS: steroid sulfatase
